# Tinnitus and Metacognitive Beliefs—Results of a Cross-Sectional Observational Study

**DOI:** 10.3390/brainsci11010003

**Published:** 2020-12-23

**Authors:** Eleonora Natalini, Alessandra Fioretti, David Riedl, Roland Moschen, Alberto Eibenstein

**Affiliations:** 1 Tinnitus Center, European Hospital, 00149 Rome, Italy; eleonora.natalini@gmail.com (E.N.); alberto.eibenstein@univaq.it (A.E.); 2 University Clinic of Medical Psychology, Medical University of Innsbruck, 6020 Innsbruck, Austria; david.riedl@tirol-kliniken.at (D.R.); roland.moschen@tirol-kliniken.at (R.M.); 3 Department of Applied Clinical and Biotechnological Sciences, University of Aquila, 67100 L’Aquila, Italy

**Keywords:** tinnitus, tinnitus distress, metacognition, worry, anxiety, depression

## Abstract

Recent research has highlighted the role of metacognitions as a moderator for psychological distress in patients with chronic diseases. The present study investigates the role of metacognitions and worry in the association between tinnitus distress, anxiety, and depression. A cross-sectional study was carried out with a sample of tinnitus-outpatients who completed the Tinnitus-Handicap Inventory, Beck Anxiety Inventory, Beck Depression Inventory, Metacognition Questionnaire-30, Penn-State-Worry-Questionnaire. Associations of metacognitions, worries, tinnitus distress, anxiety and depression were investigated using structural equation models (SEMs). A sample of *n* = 107 patients was included in the study. In the first SEM, tinnitus distress significantly predicted depression (β = 0.68, *p* < 0.001) and anxiety (β = 0.47, *p* < 0.001). In the second model, worries and meta-cognitions were added as moderators. The explained variance substantially increased for depression (46 to 53%) and anxiety (22 to 35%) and the association of tinnitus distress with depression (β = 0.57, *p* < 0.001) and anxiety was weakened (β = 0.32, *p* < 0.001). Negative beliefs significantly predicted worries (β = 0.51, *p* < 0.001) and explained 41% of its variance. A good model fit for the final model was found (comparative fit index (CFI) = 0.98; (Tucker Lewis index) TLI = 0.96; root mean square error of approximation (RMSEA) = 0.067). Anxiety and depression in tinnitus patients might be influenced by worries, which is mainly predicted by negative beliefs about uncontrollability and danger of worries. Thus, psychotherapeutic approaches focused on alterations of metacognitions in patients with tinnitus should be investigated in future studies.3 (List three to ten pertinent keywords specific to the article yet reasonably common within the subject discipline.)

## 1. Introduction

Tinnitus is a multifactorial disorder and it involves the percept of a sound or sounds, such as ringing, roaring, whistling or buzzing, in the ear or head when no external source is present [1]. Tinnitus can be linked to many different etiologies, but it in 40% of patients it is considered idiopathic, as “no known events” are associated with the tinnitus onset [2] and often the approach to tinnitus requires a multidisciplinary team to manage the complexity of the symptoms. Subjective chronic tinnitus occurs in 10 to 15% [3] of the adult population and for some patients the sound is very debilitating. Tinnitus can also coexists with hidden hearing loss [4] and may be modulated by temporo-mandibular joint and neck disorders [5,6]. In case of hearing loss and tinnitus, cochlear abnormalities are considered the initial causal source and subsequently neural changes in the central auditory system maintain the tinnitus [3,7]. The perception of tinnitus may be constant or intermittent [8]. Anxiety and depression are frequently reported by patients with tinnitus [9,10,11,12] but the psychological mechanisms that drive this association are still unclear. In patients with physical health conditions, the symptoms of anxiety and depression are common and recent reviews have highlighted the role of metacognition as a moderator for psychological distress in patients with chronic diseases [13,14]. 

The term metacognition indicates “the aspect of information processing that monitors, interprets, evaluates, and regulates the contents and processes of its organization” [15] and the Metacognitive Theory (MCT) [16] is based on the principle that the vulnerability and the extension of emotional disorders are associated with a dysfunctional style of thinking, named Cognitive Attentional Syndrome (CAS). CAS refers to repetitive negative thinking in the process of worrying and ruminating, a pattern of focusing attention on threat and dysfunctional coping strategies. Metacognitive beliefs support the CAS and they might be a relevant element associated with the process of adaptation to illness. The two main kinds of metacognitive beliefs are positive beliefs and negative beliefs. Positive beliefs about worry concern the benefits to engaging in it (e.g., “Worrying helps me cope”), while negative beliefs focus on the uncontrollability and danger of thoughts (e.g., “I could make myself sick with worrying”). Other metacognitions are cognitive confidence (e.g., “I have a poor memory”), cognitive self-consciousness (e.g., “I monitor my thoughts”) and negative beliefs about the need to control thoughts (e.g., “If I did not control a worrying thought, and then it happened, it would be my fault”).

Negative beliefs about worry seem to represent a common metacognitive pattern across pathologies such as Parkinson’s disease [17,18], cardiac illness [19] or epilepsy [20,21]. Cognitive confidence, a measure of an individual’s confidence in his own attention and memory might negatively affect coping strategies when the patient has chronic fatigue or neurological disorders, for example, feels mentally fatigued [14].

Previous studies have not investigated the role of metacognitive beliefs in tinnitus distress, but in a single group study [22] MCT was used to treat tinnitus suffers. Results indicated a reduction in the perception of the tinnitus, anxiety, and in the significance of annoying thinking. In addition, Caldirola and colleagues [23] examined the function of worry in patients with chronic tinnitus. In this article tinnitus handicap, anxiety and depressive symptoms were significantly associated with proneness to worry (linear regression models, *p* < 0.01).

This evidence suggests the need to analyze metacognitive beliefs and the tendency to worry in patients with tinnitus to improve treatments. The aim of the study was to investigate the role of metacognitions and worry in the association between tinnitus distress and the onset of anxiety and depression.

## 2. Materials and Methods

### 2.1. Sample and Setting

A sample of *n* = 107 consecutive outpatients was included in this cross-sectional study. Patients were evaluated between April 2018 and April 2019 at the Tinnitus Center of European Hospital (Rome). Inclusion criteria were (a) presence of tinnitus, (b) age over 18 years, (c) speaking Italian fluently and (d) absence of cognitive impairment or major psychiatric/neurological disorders (schizophrenia, Alzheimer’s disease, Parkinson’s disease). All patients were evaluated with otorhinolaryngological and psychological examination, audiometry and questionnaires as part of the routine clinical practice. Based on the duration of tinnitus, patients were classified as with acute tinnitus (<3 months), subacute tinnitus (3–6 months) and chronic tinnitus (>6 months). The study was conducted in accordance with the Declaration of Helsinki. The protocol of the study was approved by the Ethics Committee of University of L’Aquila (ID number 42:2020, protocol number 128446). Informed consent was obtained by every patient before filling out the questionnaires.

### 2.2. Measures

#### 2.2.1. Tinnitus Sample Case History (TSCH)

The TSCH is a standardized questionnaire of 35 items to collect sociodemographic and clinical data in tinnitus research [24]. The Italian version of the TSCH was used to collect significant information, such as patient background (i.e., age, gender), tinnitus characteristics (i.e., loudness, pitch, perception at onset, tinnitus location, percentage of awake time aware of tinnitus) and related medical conditions (i.e., hearing impairment, noise annoyance, vertigo/dizziness, headache, Temporomandibular joint disorders, cervical disorders).

#### 2.2.2. Tinnitus Handicap Inventory (THI)

The THI [25] is a self-report questionnaire widely used in tinnitus research to assess the impact of tinnitus in daily life. It consists of 25 items and 3 subscales (functional, emotional and catastrophic). The Italian THI version has a good reliability (α = 0.94) and validity [26] and was used to assess tinnitus severity graded from slight (grade 1) to catastrophic (grade 5). A THI score > 36 was considered to indicate a decompensated tinnitus [27,28].

#### 2.2.3. Beck Anxiety Inventory (BAI)

The BAI [29,30] is a self-administered tool of 21-items to assess the severity of anxiety over the last week. Every item can be rated on a 4-point scale ranging from 0 (“not at all”) to 3 (“severely”). Based on the final total score, anxiety can be classified as minimal (0–7), mild (8–15), moderate (16–25), and severe (26–63).

#### 2.2.4. Beck Depression Inventory (BDI)

The BDI-II [30,31,32] is a self-report instrument of 21 items to assess depressive symptoms over the previous two weeks. Based on the final total score, depression can be classified as minimal (0–13), mild (14–19), moderate (20–28) or severe (29–63).

#### 2.2.5. Metacognition Questionnaire-30 (MCQ-30)

The MCQ-30 [33,34] is a 30-item questionnaire designed to assess levels of dysfunctional metacognitions. The MCQ-30 contains 5 subscales: (1) positive beliefs about worry (pos); (2) negative beliefs about the controllability of thoughts and danger of worry (neg); (3) cognitive confidence (CC); (4) beliefs about the need to control thoughts (NC); and (5) cognitive self-consciousness (CSC). Higher levels of dysfunctional metacognitions are detected by higher scores on the total score as well as the subscales.

#### 2.2.6. Penn State Worry Questionnaire (PSWQ)

The PSWQ [35,36] is a 16-item self-administered scale to measure worry. The items are scored on a 5-point Likert-type scale (1—not at all typical of me, to 5—very typical of me). Based on the final total score, the patients’ worries can be classified as low (16–39), moderate (40–59) and high (60–80).

#### 2.2.7. Tonal Audiometry

The presence of hearing loss was investigated with tonal audiometry. Pure tone audiometry was carried out using a clinical audiometer (Madsen Itera II, GN Otometrics) and patients were evaluated into an audiological cabin. Normal hearing was defined by threshold < 25 dB HL in all frequencies tested between 250 Hz and 8.000 Hz.

### 2.3. Statistical Analyses

**Figure 1:**
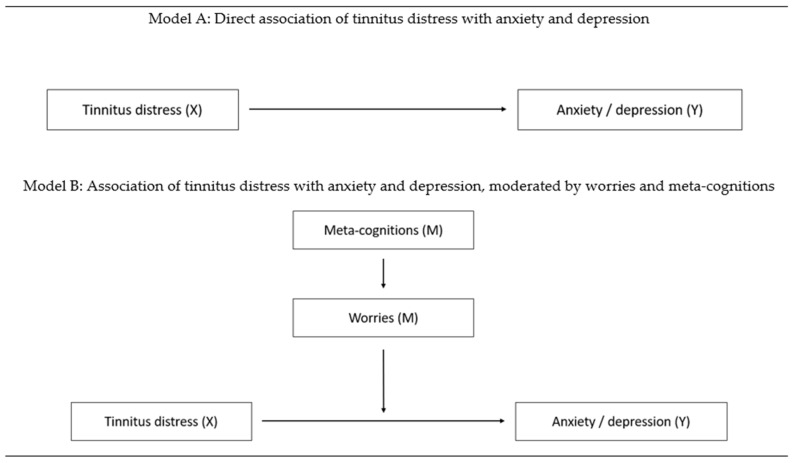
Structural equation models to test the moderating effect of worries and meta-cognitions on the relationship of tinnitus distress with anxiety and depression. Model A depicts the direct association of tinnitus distress with the dependent variables (anxiety, depression). Model B depicts the model with worries as a moderator of the association of tinnitus distress with the dependent variables (anxiety, depression) and meta-cognitions as predictors for worries.

Demographics for the sample are presented with means and standard deviations (SD). The relationship of tinnitus distress, meta-cognitions, and depression, anxiety and worries were investigated with two structural equation models (see Figure 1). In model A the direct influence of tinnitus distress on depression and anxiety was tested. In model B, worries were added to the model as moderators for this relationship and meta-cognitions were added as predictors for worries. The association of the five assessed meta-cognitions with tinnitus distress, anxiety, depression and worries were tested with Pearson correlation coefficients prior to inclusion to the model. Meta-cognitions were added to the model if a significant association was found with at least one of the dependent variables. Pearson’s chi-squared test (χ^2^), the comparative fit index (CFI) and root mean square error of approximation (RMSEA) with lower and higher bounds of the 95% confidence interval (CI) were calculated to determine the model’s goodness of fit. To evaluate whether the empirical data fit closely with the theoretical model, the *p*-value of Close Fit (PCLOSE) was calculated based on the RMSEA values, with values of *p* > 0.05 indicating close fit and *p* < 0.05 indicating worse than close model fit. Acceptable goodness of fit was defined as RMSEA values of < 0.08 and CFI values > 0.90. *p*-values < 0.05 (two-sided) were considered statistically significant. Statistical analyses were performed with IBM SPSS (v22.0) and SPSS AMOS (v24.0).

## 3. Results

A total of *n* = 107 patients were included in the analyses (60.7% male). A total of 77.1% of patients reported chronic tinnitus and 63.6% had a hearing loss. Details on patient characteristics are reported in Table 1. Based on the total THI score, 19.6% of the patients were classified as very mild (grade I), 28.0% mild (grade II), 29.0% moderate (grade III), 15.9% severe (grade IV), and 7.5% very severe (grade V). Based on the cut-off of 36 on the THI, 52.3% of the patients reported a decompensated tinnitus.

Regarding depression, the majority of the present sample reported no or minimal symptoms (*n* = 75; 70.1%), while the remaining patients showed mild (*n* = 17; 15.9%), moderate (*n* = 12; 11.2%) and severe (*n* =3; 2.8%) depressive symptoms. Prevalence rates for anxiety were slightly higher with approximately half of the patients showing no or mild symptoms (*n* = 55; 51.4%), while the remaining patients showed mild (*n* = 29; 27.1%), moderate (*n* = 16; 15.0%) and severe (*n* = 7; 6.5%) anxiety symptoms. Overall, women reported higher values for anxiety (12.6 vs. 8.1 points; *t* = 2.6, *p* = 0.010), depression (12.5 vs. 9.1 points; *t* = 2.4; *p* = 0.019) and worries (51.0 vs. 45.2 points; *t* = 2.4; *p* = 0.015).

### 3.1. Meta-Cognitions in Patients with Tinnitus

The highest correlations between MCQ-subscales were found for negative beliefs about the controllability of thoughts and danger of worry (MCQ neg) with beliefs about the need to control thoughts (MCQ NC) and with cognitive self-consciousness (MCQ CSC). No association was found between cognitive confidence (MCQ CC) and negative beliefs about the controllability of thoughts and danger of worry (MCQ neg), beliefs about the need to control thoughts (MCQ NC), and cognitive self-consciousness (MCQ CSC) (Table 2). No gender differences were found for the MCQ-30 subscales, except for the need to control thoughts, with males scoring higher on this subscale (11.3 vs. 9.9 points; *t* = 2.03; *p* = 0.045). None of the MCQ-30 subscales were associated with patients age, subjective tinnitus loudness, or awareness of tinnitus (all *p* > 0.05). However, both the negative beliefs about uncontrollability and danger of worries (*r* = 0.26; *p* = 0.016) as well as the need for control (*r* = 0.26; *p* = 0.016) were significantly associated with a higher level of annoyance be the ear noise.

### 3.2. The Association of Tinnitus Distress with Meta-Cognitions, Anxiety, Depression and Worry

The results of our study evidenced that higher levels of tinnitus distress were significantly associated with negative beliefs about the controllability of thoughts and danger of worry and beliefs about the need to control thoughts. Anxiety, depression, and worries were significantly associated with negative beliefs about the controllability of thoughts and danger of worry, cognitive confidence and beliefs about the need to control thoughts. For worries, an additional positive correlation was observed with higher cognitive self-consciousness. Overall, strongest correlations with tinnitus distress and anxiety, depression and worries were found for negative beliefs about the controllability of thoughts and danger of worry. For details see Table 3.

### 3.3. Direct Association of Tinnitus Distress with Depression and Anxiety

**Figure 2:**
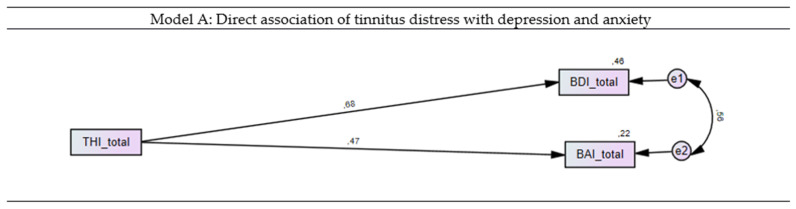
Structural equation model (SEM) for the direct influence of tinnitus distress on depression and anxiety. Rectangles represent variables and circles represent error terms (e). Arrows indicate paths, double arrows represent covariances. Numbers next to arrows in the model represent statistically significant standardized estimates, numbers next to factors represent the R^2^, i.e., the explained variance.

In the first step, the direct associations of tinnitus distress with depression and anxiety were investigated by calculation of a SEM. Tinnitus distress significantly predicted depression (*p* < 0.001, β = 0.68) and anxiety (*p* < 0.001, β = 0.47) and explained 46% and 22% of the variance, respectively. Strong intercorrelations were found between depression and anxiety (*r* = 0.56) (Figure 2).

### 3.4. Meta-Cognitions and Worries as Moderators for the Association of Tinnitus Distress with Anxiety and Depression

In the second step, the PSWQ total score and MCQ-30 subscales were added to the model as moderators. As shown in Table 2, significant correlations for four of the five MCQ-30 subscales with anxiety depression and worries were found, namely positive beliefs about worry, negative beliefs about the controllability of thoughts and danger of worry, cognitive confidence, beliefs about the need to control thoughts and cognitive self-consciousness with the factors of model one were found. Thus, four of the five MCQ-30 subscales were added to the model.

**Figure 3:**
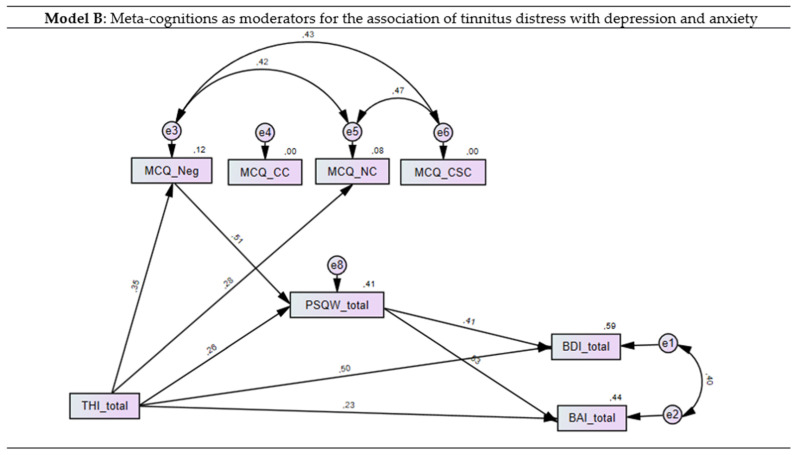
Structural equation model (SEM) for the moderated influence of tinnitus distress on depression and anxiety. Worries were added to the model as moderators for the two dependent variables and meta-cognitions as predictors for worries. Pos = positive beliefs about worry; neg = negative beliefs about the controllability of thoughts and danger of worry; CC = cognitive confidence; NC = beliefs about the need to control thoughts; CSC = cognitive self-consciousness. Rectangles represent variables and circles represent error terms (e). Numbers next to arrows in the model represent statistically significant standardized estimates, numbers next to factors represent the R^2^, i.e., the explained variance.

The overall explained variance substantially increased for depression (59%) and anxiety (44%) and the direct association of tinnitus distress with both depression (*p* < 0.001; β = 0.57) and anxiety (*p* < 0.001, β = 0.32) was weakened. Negative beliefs (β = 0.51, *p* < 0.001) significantly predicted worries and explained 41% of the variance. After exclusion of non-significant paths, a good model fit was found for the final model (χ^2^ = 23.7, *p* = 0.09; CMIN/DF = 1.48; CFI = 0.98; TLI = 0.96; RMSEA = 0.067, 95%CI 0.00–0.12; PCLOSE = 0.28) (Figure 3).

## 4. Discussion

Anxiety and depression are frequently reported by patients with tinnitus. The psychological mechanisms that drive the association between tinnitus distress and anxiety and depression are still unclear. Many tinnitus pathophysiology models have been developed to investigate the mechanisms of tinnitus awareness and distress, such as the neurophysiological model, the habituation theory, the fear-avoidance model and the cognitive model [1]. The Cognitive Behavioral Model of Tinnitus proposes the interaction between tinnitus and negative thoughts, negative emotions, attention and monitoring, safety behavior, and beliefs [37]. The aim of the present study was to investigate the role of metacognition and worry in the association between tinnitus distress, anxiety and depression. As hypothesized, we found that metacognitive beliefs and worry predicted the development of anxiety and depression in patients with tinnitus. Studies using connectivity neuroimaging techniques like diffusion MRI and resting-state fMRI have been devoted to exploring the neuroimaging relationship between tinnitus and auditory–limbic interactions [38]. A strong connectivity between the ventral and dorsal prefrontal cortical network was recently demonstrated by fMRI in the regulation of action selection based on metacognition but this has not yet been shown in tinnitus research [39].

Almost a third of the patients in our study reported depressive symptoms while almost half of them reported relevant levels of anxiety. In accordance with previous studies [9,10,11,12], increased levels of tinnitus distress were associated with higher levels of depression and anxiety. Additionally, women reported higher values in anxiety and depression which may confirm a specific gender effect for the assessed psychological comorbidities [9,40,41].

Higher levels of tinnitus distress were associated with negative beliefs about the controllability of thought, as well as with a higher need to control thoughts. Anxiety, depression, and worries were significantly associated with negative metacognitive beliefs, cognitive confidence and beliefs about the need to control thoughts. For worries, an additional positive correlation, was observed with higher cognitive self-consciousness. Our results confirmed the prominent role of metacognitive beliefs in the CAS maintaining psychological disorders [16].

In our study we also tested the hypothesis that meta-cognitions influence the tendency for worries, which in itself may be a moderator for the relationship of tinnitus distress with anxiety and depression. As hypothesized, the inclusion of worries led to a substantial increase in the explained variance in both depression and anxiety. Furthermore, interesting data were reported in model B of the two structural equation models, where the PSWQ total score and MCQ-30 subscales were added as moderators. The overall explained variance substantially increased for depression (59%) and anxiety (44%) and the direct association of tinnitus distress with both depression and anxiety was significantly weakened after inclusion of worries, indicating that worries might indeed moderate the relationship between tinnitus distress, depression and anxiety. Worries on the other hand were significantly predicted by meta-cognitions which explained 41% of the variance of the variable worries. However, this association was mainly caused by the factor negative beliefs about the uncontrollability and danger of worries, while the other included MCQ-30 subscales were not significant in the structural equation model. Our results thus confirmed that worry and metacognitive beliefs are related to the development and maintenance of anxiety and depression symptoms [42,43] and that negative beliefs are a significant predictor of these symptoms in different illness types [17,44,45].

Tinnitus is usually considered chronic after 6 months [1]. In many cases the natural history of tinnitus is the habituation to the auditory sensation, but in approximately 20% of patients the tinnitus is considered bothersome [46]. Bothersome tinnitus affects sleep [47], concentration and psychological stability [40,41,48,49,50,51]. In a previous study, Budd and Pugh [52] reported that the adaptation or habituation to tinnitus may occur over time as a result of avoidance of maladaptive coping strategies, rather than due to the patients active use of effective coping strategies. Worry is a kind of maladaptive coping strategy that maintains attention on threats, and it has an important function in the CAS [16]. As highlighted in the present study, worries are significantly associated with increased anxiety and depression in tinnitus patients and negative beliefs may be a driving factor in this association. The results thus confirmed that the importance of dysfunctional metacognitive beliefs and the tendency to worry are relevant factors associated within the process of adapting to a chronic condition illness, which has also been highlighted in recent studies on psychological distress in patients with chronic diseases [13,14].

As for now, the standard technique of Cognitive Behavioral Therapy (CBT) [53] focusses on thought content instead of on the thinking process. However, psychological interventions centered on the MCT [16] could facilitate treatments for patients with tinnitus using specific techniques as Detached Mindfulness and Attentional Training Technique (ATT) [54] to manage worry. These techniques develop the activation of metacognitive knowledge, attentional flexibility and a decentered relationship with thoughts. Patients can learn to observe their thoughts and feelings and experience them, without automatically identifying with them. In particular, the ATT could be adapted to help patients shift attention from tinnitus to other stimuli. The finding that negative thoughts and dysfunctional cognitions appears to be linked to a worse experience of tinnitus was recently investigated by other authors using the Tinnitus Cognitions Questionnaire (TCQ) [55] and the Tinnitus Cognitions Scale (T-Cog) [56]. In the present study we focused our attention on the thought process using the MCQ to demonstrate that worry and metacognition play a fundamental role in maintaining the disorder even in patients with tinnitus.

This study has some limitations. First, we could not find a significant difference between worry and metacognition in acute and chronic because we evaluated a high number of patients with chronic tinnitus (77.1%). Second, our sample is small and we did not investigate the presence of other medical comorbidities (diabetes, insomnia, migraine, hypertension) and tinnitus pitch and loudness. Third, we did not analyze the results based on particular tinnitus subtypes. Additionally, the study was conducted as a cross-sectional observational study and thus no causal conclusions can be drawn from the study. However, the observed correlations give indications for future research and allow valuable theoretical conclusions.

## 5. Conclusions

Anxiety and depression in tinnitus patients might be influenced by worries, which are mainly predicted by negative beliefs about uncontrollability and danger of worries. Thus, psychotherapeutic approaches focused on alterations of metacognitions in patients with tinnitus should be investigated in future studies.

## Figures and Tables

**Table 1:** Sociodemographic and clinical data.

	Mean	(SD)
Age	49.1	(13.9)
Tinnitus loudness (0–100)	52.2	(24.7)
Awareness of tinnitus ^a^	79.8	(28.0)
Annoyed by tinnitus ^a^	49.8	(32.7)
	*N*	*%*
Tinnitus: family history	31	29.0%
Missing	5	4.7%
Duration tinnitus		
<3 months	17	15.9%
3–6 months	7	6.7%
>6 months	81	77.1%
Missing	2	1.9%
Tinnitus perception at onset		
Gradual	71	78.9%
Abrupt	19	21.1%
Missing	17	15.9%
Pulsating tinnitus	18	17.1%
Missing	2	1.9%
Tinnitus location		
right ear	15	14.2%
left ear	23	21.7%
both ears	62	58.5%
inside the head	6	5.7%
Missing	1	0.9%
Tinnitus loudness varies from day to day	34	32.4%
Missing	2	1.9%
Tinnitus manifestation		
Intermittent	23	22.5%
Constant	79	77.5%
Missing	5	4.7%
Tinnitus sound		
Tone	49	48.5%
Noise	24	23.8%
Crickets	10	9.9%
Other	18	17.8%
Missing	6	5.6%
Objective hearing problem ^b^	68	63.6%
Missing		
Subjective hearing problem	63	61.8%
Missing	5	4.7%
Hyperacusis	67	66.3%
Missing	6	5.6%
Headache	65	61.9%
Missing	2	1.9%
Vertigo/dizziness	79	76.0
Missing	3	2.8%
Temporomandibular disorder	34	33.7%
Missing	6	5.6%
Neck pain	60	57.7%
Missing	3	2.8%
Other pain	27	27.6%
Missing	9	8.4%

^a^ Percentage of awake time; N: number of patients; ^b^ based on tonal audiometry (250–8.000 Hz).

**Table 2:** Correlations of MCQ-30 subscales.

	MCQ Pos	MCQ Neg	MCQ CC	MCQ NC	MCQ CSC
MCQ pos	-	0.26 **	0.26 **	0.34 ***	0.39 ***
MCQ neg	-	-	0.11	0.49 ***	0.43 ***
MCQ CC	-	-	-	0.094	0.085
MCQ NC	-	-	-	-	0.47 ***
MCQ CSC	-	-	-	-	-

** *p* < 0.01, *** *p* < 0.001; MCQ = Metacognition Questionnaire; pos = positive beliefs about worry; neg = negative beliefs about the controllability of thoughts and danger of worry; CC = cognitive confidence; NC = beliefs about the need to control thoughts; CSC = cognitive self-consciousness.

**Table 3:** Correlations of tinnitus distress and meta-cognitions, anxiety, depression and worries.

	MCQ Pos	MCQ Neg	MCQ CC	MCQ NC	MCQ CSC
THI total score	0.13	0.38 ***	0.15	0.32 **	0.10
BDI	−0.16	0.51 ***	0.23 *	0.29 **	0.19
BAI	0.02	0.50 ***	0.33 **	0.26 **	0.15
PSWQ total score	0.11	0.61 ***	0.20 *	0.31 **	0.36 ***

* *p* < 0.05, ** *p* < 0.01, *** *p* < 0.001; THI = Tinnitus Handicap Inventory; MCQ = Metacognition Questionnaire; pos = positive beliefs about worry; neg = negative beliefs about the controllability of thoughts and danger of worry; CC = cognitive confidence; NC = beliefs about the need to control thoughts; CSC = cognitive self-consciousness; PSWQ = Penn State Worry Questionnaire; BDI = Beck Depression Inventory; BAI = Beck Anxiety Inventory.

## Data Availability

The data presented in this study are available on request from the corresponding author A.F. The data are not publicly available since the consent of the patients was not obtained.

## References

[1] Cima R.F.F., Mazurek B., Haider H., Kikidis D., Lapira A., Norena A., Hoare D.J. (2019). A multidisciplinary European guideline for tinnitus: Diagnostics, assessment, and treatment. HNO.

[2] Henry J.A., Dennis K.C., Schechter M.A. (2005). General review of tinnitus: Prevalence, mechanisms, effects, and management. JSLHR.

[3] Baguley D., McFerran D., Hall D. (2013). Tinnitus. Lancet.

[4] Kara E., Aydin K., Akbulut A.A., Karakol S.N., Durmaz S., Yener H.M., Gozen E.D., Kara H. (2020). Assessment of Hidden Hearing Loss in Normal Hearing Individuals with and Without Tinnitus. J. Int. Adv. Otol..

[5] Edvall N.K., Gunan E., Genitsaridi E., Lazar A., Mehraei G., Billing M., Tullberg M., Bulla J., Whitton J., Canlon B. (2019). Impact of Temporomandibular Joint Complaints on Tinnitus-Related Distress. Front. Neurosci..

[6] Haider H.F., Hoare D.J., Costa R.F.P., Potgieter I., Kikidis D., Lapira A., Nikitas C., Caria H., Cunha N.T., Paço J.C. (2017). Pathophysiology, Diagnosis and Treatment of Somatosensory Tinnitus: A Scoping Review. Front. Neurosci..

[7] Shore S.E., Roberts L.E., Langguth B. (2016). Maladaptive plasticity in tinnitus--triggers, mechanisms and treatment. Nat. Rev. Neurol..

[8] Schlee W., Pryss R.C., Probst T., Schobel J., Bachmeier A., Reichert M., Langguth B. (2016). Measuring the Moment-to-Moment Variability of Tinnitus: The TrackYourTinnitus Smart Phone App. Front. Aging Neurosci..

[9] Pattyn T., Van Den Eede F., Vanneste S., Cassiers L., Veltman D.J., Van De Heyning P., Sabbe B.C.G. (2016). Tinnitus and anxiety disorders: A review. Hear. Res..

[10] Bhatt J.M., Bhattacharyya N., Lin H.W. (2017). Relationships between tinnitus and the prevalence of anxiety and depression. Laryngoscope.

[11] Trevis K.J., McLachlan N.M., Wilson S.J. (2018). A systematic review and meta-analysis of psychological functioning in chronic tinnitus. Clin. Psychol. Rev..

[12] Salazar J.W., Meisel K., Smith E.R., Quiggle A., Mccoy D.B., Amans M.R. (2019). Depression in Patients with Tinnitus: A Systematic Review. Otolaryngol. Head Neck Surg..

[13] Capobianco L., Faija C., Husain Z., Wells A. (2020). Metacognitive beliefs and their relationship with anxiety and depression in physical illnesses: A systematic review. PLoS ONE.

[14] Lenzo V., Sardella A., Martino G., Quattropani M.C. (2020). A Systematic Review of Metacognitive Beliefs in Chronic Medical Conditions. Front. Psychol..

[15] Wells A., Purdon C.L. (1999). Metacognition and cognitive-behaviour therapy: A special issue. Clin. Psychol. Psychother..

[16] Wells A. (2000). Emotional Disorders and Metacognition: Innovative Cognitive Therapy.

[17] Allott R., Wells A., Morrison A.P., Waller R. (2005). Distress in Parkinson’s disease: Contributions of disease fac- tors and metacognitive style. Br. J. Psychiatry.

[18] Brown R.G., Fernie B.A. (2015). Metacognitions, anxiety, and distress related to motor fluctuations in Parkinson’s disease. J. Psychosom. Res..

[19] Compare A., Brugnera A., Spada M.M., Zarbo C., Tasca G.A., Sassaroli S., Caselli G., Ruggiero G.M., Wittstein I. (2018). The Role of Emotional Competence in Takotsubo Cardiomyopathy. Psychosom. Med..

[20] Fisher P.L., Noble A.J. (2017). Anxiety and depression in people with epilepsy: The contribution of metacognitive beliefs. Seizure.

[21] Fisher P.L., Reilly J., Noble A. (2018). Metacognitive beliefs and illness perceptions are associated with emotional distress in people with epilepsy. Epilepsy Behav..

[22] Ferraro L., Barbera D.L., Messina A., Galioto S., Marinaro A., Caruso C., Rizzo R., Cascia C.L. (2019). Metacognitive Therapy in Patients with Tinnitus: A Single Group Study. J. Contemp. Psychother..

[23] Caldirola D., Teggi R., Dacco S., Sangiorgio E., Bussi M., Perna G. (2016). Role of worry in patients with chronic tinnitus and sensorineural hearing loss: A preliminary study. Eur. Arch. Otorhinolaryngol..

[24] Langguth B., Goodey R., Azevedo A., Bjorne A., Cacace A., Crocetti A., Del Bo L., De Ridder D., Diges I., Elbert T. (2007). Consensus for tinnitus patient assessment and treatment outcome measurement: Tinnitus Research Initiative meeting, Regensburg, July 2006. Prog. Brain Res..

[25] Newman C.W., Jacobson G.P., Spitzer J.B. (1996). Development of the Tinnitus Handicap Inventory. Arch. Otolaryngol. Head Neck Surg..

[26] Passi S., Ralli G., Capparelli E., Mammone A., Scacciatelli D., Cianfrone G. (2008). The THI questionnaire: Psychometric data for reliability and validity of the Italian version. Int. Tinnitus J..

[27] Salviati M., Macri F., Terlizzi S., Melcore C., Provenzano A., Capparelli E., Altissimi G., Cianfrone G. (2013). The Tinnitus Handicap Inventory as a screening test for psychiatric comorbidity in patients with tinnitus. Psychosomatics.

[28] Altissimi G., Salviati M., Turchetta R., Orlando M.P., Greco A., De Vincentiis M., Ciofalo A., Marinelli C., Testugini V., Mazzei F. (2016). When alarm bells ring: Emergency tinnitus. Eur. Rev. Med. Pharmacol. Sci..

[29] Beck A.T., Epstein N., Brown G., Steer R.A. (1988). An inventory for measuring clinical anxiety: Psychometric properties. J. Consult. Clin. Psychol..

[30] Sica C., Ghisi M., Lange M.A. (2007). The Italian versions of the Beck Anxiety Inventory and the Beck Depression Inventory-II: Psychometric properties and discriminant power. Leading-Edge Psychological Tests and Testing Research.

[31] Beck A.T., Steer R.A., Brown G.K. (1996). Manual for the Beck Depression Inventory–II.

[32] Montano A., Flebus G.B. (2006). Presentation of the Beck Depression Inventory—Second edition (BDI-II): Confirmation of bifactorial structure in a sample of the Italian population. Psicoter. Cogn. Comport..

[33] Wells A., Cartwright-Hatton S. (2004). A short form of the metacognitions questionnaire: Properties of the MCQ-30. Behav. Res. Ther..

[34] Quattropani M.C., Lenzo V., Mucciardi M., Toffle M.E. (2014). Psychometric properties of the Italian version of the Short Form of the Metacognitions Questionnaire (MCQ-30). BPA.

[35] Meyer T.J., Miller M.L., Metzger R.L., Borkovec T.D. (1990). Development and validation of the Penn State Worry Questionnaire. Behav. Res. Ther..

[36] Morani S., Pricci D., Sanavio E. (1999). “Penn State Worry Questionnaire” e “Worry Domains Questionnaire”. Presentazione delle versioni italiane ed analisi della fedeltà. Psicoter. Cogn. Comport..

[37] McKenna L., Handscomb L., Hoare D.J., Hall D.A. (2014). A scientific cognitive-behavioral model of tinnitus: Novel conceptualizations of tinnitus distress. Front. Neurol..

[38] Leaver A.M., Seydell-Greenwald A., Rauschecker J.P. (2016). Auditory-limbic interactions in chronic tinnitus: Challenges for neuroimaging research. Hear. Res..

[39] Yuki S., Nakatani H., Nakai T., Okanoya K., Tachibana R.O. (2019). Regulation of action selection based on metacognition in humans via a ventral and dorsal medial prefrontal cortical network. Cortex.

[40] Strumila R., Lengvenyte A., Vainutiene V., Lesinskas E. (2017). The role of questioning environment, personality traits, depressive and anxiety symptoms in tinnitus severity perception. Psychiatr. Q..

[41] Ziai K., Moshtaghi O., Mahboubi H., Djalilian H.R. (2017). Tinnitus Patients Suffering from Anxiety and Depression: A Review. Int. Tinnitus J..

[42] Huntley C.D., Fisher P.L. (2016). Examining the role of positive and negative metacognitive beliefs in depression. Scand. J. Psychol..

[43] Ryum T., Kennair L., Hjemdal O., Hagen R., Halvorsen J.Ø., Solem S. (2017). Worry and Metacognitions as Predictors of Anxiety Symptoms: A Prospective Study. Front. Psychol..

[44] Cook S.A., Salmon P., Dunn G., Holcombe C., Cornford P., Fisher P. (2015). The association of metacognitive beliefs with emotional distress after diagnosis of cancer. Health Psychol..

[45] Purewal R., Fisher P.L. (2018). The contribution of illness perceptions and metacognitive beliefs to anxiety and depression in adults with diabetes. Diabetes Res. Clin. Pract..

[46] Tunkel D.E., Bauer C.A., Sun G.H., Rosenfeld R.M., Chandrasekhar S.S., Cunningham E.R., Archer S.M., Blakley B.W., Carter J.M., Granieri E.C. (2014). Clinical practice guideline: Tinnitus. Otolaryngol. Head Neck Surg..

[47] Aazh H., Baguley D.M., Moore B.C.J. (2019). Factors Related to Insomnia in Adult Patients with Tinnitus and/or Hyperacusis: An Exploratory Analysis. J. Am. Acad. Audiol..

[48] Meric C., Gartner M., Collet L., Chery-Croze S. (1998). Psychopathological profile of tinnitus sufferers: Evidence concerning the relationship between tinnitus features and impact on life. Audiol. Neurootol..

[49] Durai M., Searchfield G. (2016). Anxiety and depression, personality traits relevant to tinnitus: A scoping review. Int. J. Audiol..

[50] Gomaa M.A., Elmagd M.H., Elbadry M.M., Kader R.M. (2014). Depression, Anxiety and Stress Scale in patients with tinnitus and hearing loss. Eur. Arch. Otorhinolaryngol..

[51] Udupi V.A., Uppunda A.K., Mohan K.M., Alex J., Mahendra M.H. (2013). The relationship of perceived severity of tinnitus with depression, anxiety, hearing status, age and gender in individuals with tinnitus. Int. Tinnitus J..

[52] Budd R.J., Pugh R. (1996). Tinnitus coping style and its relationship to tinnitus severity and emotional distress. J. Psychosom. Res..

[53] Beck A.T. (1976). Cognitive Therapy and the Emotional Disorders.

[54] Wells A. (2009). Metacognitive Therapy for Anxiety and Depression.

[55] Handscomb L.E., Hall D.A., Shorter G.W., Hoare D.J. (2017). Positive and Negative Thinking in Tinnitus: Factor Structure of the Tinnitus Cognitions Questionnaire. Ear Hear..

[56] Conrad I., Kleinstäuber M., Jasper K., Hiller W., Andersson G., Weise C. (2015). The Role of Dysfunctional Cognitions in Patients with Chronic Tinnitus. Ear Hear..

